# Reintroduction of rare arable plants by seed transfer. What are the optimal sowing rates?

**DOI:** 10.1002/ece3.2303

**Published:** 2016-07-12

**Authors:** Marion Lang, Julia Prestele, Christina Fischer, Johannes Kollmann, Harald Albrecht

**Affiliations:** ^1^ Restoration EcologyDepartment of Ecology and Ecosystem ManagementTechnische Universität MünchenFreisingGermany; ^2^ Institute for Organic Farming, Soil and Resource ManagementBavarian State Research Centre for AgricultureFreisingGermany

**Keywords:** Agro‐biodiversity, crop yield loss, density effects, establishment success, rare weeds, Red List species, restoration

## Abstract

During the past decades, agro‐biodiversity has markedly declined and some species are close to extinction in large parts of Europe. Reintroduction of rare arable plant species in suitable habitats could counteract this negative trend. The study investigates optimal sowing rates of three endangered species (*Legousia speculum‐veneris* (L.) Chaix, *Consolida regalis* Gray, and *Lithospermum arvense* L.), in terms of establishment success, seed production, and crop yield losses.
A field experiment with partial additive design was performed in an organically managed winter rye stand with study species added in ten sowing rates of 5–10,000 seeds m^−2^. They were sown as a single species or as a three‐species mixture (pure vs. mixed sowing) and with vs. without removal of spontaneous weeds. Winter rye was sown at a fixed rate of 350 grains m^−2^. Performance of the study species was assessed as plant establishment and seed production. Crop response was determined as grain yield.
Plant numbers and seed production were significantly affected by the sowing rate, but not by sowing type (pure vs. mixed sowing of the three study species), and weed removal. All rare arable plant species established and reproduced at sowing rates >25 seeds m^−2^, with best performance of *L. speculum‐veneris*. Negative density effects occurred to some extent for plant establishment and more markedly for seed production.
The impact of the three study species on crop yield followed sigmoidal functions. Depending on the species, a yield loss of 10% occurred at >100 seeds m^−2^.
*Synthesis and applications*: The study shows that reintroduction of rare arable plants by seed transfer is a suitable method to establish them on extensively managed fields, for example, in organic farms with low nutrient level and without mechanical weed control. Sowing rates of 100 seeds m^−2^ for *C. regalis* and *L. arvense*, and 50 seeds m^−2^ for *L. speculum‐veneris* are recommended, to achieve successful establishment with negligible crop yield losses.

## Introduction

Arable plants growing spontaneously within cultivated crops provide benefits for many organisms and support several ecosystem functions, thus essentially contributing to agro‐biodiversity (Marshall et al. 2003). During the past decades, these species have been negatively affected by the intensification of management, and a strong decline in plant diversity has been reported (reviewed in Storkey et al. 2012). In Germany, 35% of the species closely adapted to arable farming are listed in the Red Data Book of threatened plants (Korneck et al. 1996). In the United Kingdom, Still and Byfield (2007) consider the arable flora to be “the most threatened group of plants in Britain today.” While most of these reports reflect conditions in Europe, recent studies increasingly indicate global patterns of this plant diversity loss (Bambaradeniya et al. 2004; Yamada et al. 2007; Türe and Böcük 2008; Nowak et al. 2014).

Plant populations within agro‐ecosystems are mainly driven by farming disturbance, resource supply, and crop competition which occur in deterministic mosaic cycles. This means that habitat quality regularly changes spatially and temporally. (Kleyer et al. 2007). After soil tillage, lack of competition and good resource supply periodically provide suitable conditions for seedling establishment. However, in intensively managed fields, herbicides are applied mainly during the early growth stages and delete the early germinating plants. Consequently, winter annual species are most negatively affected and thus most endangered among arable plants (Albrecht 2003).

Reduced land‐use intensity provides favourable conditions for rare arable plants, as seen in less developed regions and under organic farming in developed regions (Cambecèdes 2011; Richner et al. 2015). The European Union agro‐environmental schemes, which have been developed to conserve and increase species diversity of arable land (Uthes and Matzdorf 2013), cannot counteract the losses of arable plant communities. This is because wildflower programms, where species mixtures are sown in species‐poor fields to benefit wildlife (Dicks et al. 2013), do not contain arable plants. Unsprayed field margins, on the other hand, benefit arable plant communities (Schumacher 1980), but noxious weeds such as *Cirsium arvense* or highly competitive grasses cause frequent problems (Wicke 1998). A further approach is the conservation of sites with an outstanding spectrum of threatened species in fields with low management intensity (Meyer et al. 2010; Cambecèdes 2011). As this approach necessitates regular efforts to maintain threatened populations, these conservation sites are rare and may not suffice to effectively reduce the extinction risk for small and isolated populations. Therefore, restoration approaches which integrate arable species conservation into regular farming practice need to be developed. Organic fields offer suitable habitats for species‐rich arable plant communities (Albrecht and Mattheis 1998; Bengtsson et al. 2005; Hole et al. 2005; Romero et al. 2008), as the use of synthetic nitrogen and herbicides is prohibited. However, arable plants are almost absent from many farms, due to previous intense management and their dispersal limitation. Rotchés‐Ribalta et al. (2015a) argue that the occurrence of rare arable species on organic farms is less determined by farming practice but dependant on local species pools set by the field history. At sites where the species pool is missing, the reintroduction of plants provides an opportunity to conserve plant diversity. Besides suitable site and management conditions, sowing rates of the study species may play an important role for a successful reintroduction by seed transfer, as density‐dependent effects, triggered by intra‐ and interspecific competition, control population dynamics (Cousens and Mortimer 1995). There is little research on density effects of arable plants considering the whole life cycle in the presence of a crop (Cousens and Mortimer 1995).

So far, research on weed competition has focused on species that are well known for their negative impact on crop yields (Zimdahl 2004), while less competitive species have been studied rarely. Calculating response functions of crop yield and weed density in 12 annual species, Wilson and Wright (1990) mainly found limited competitive ability of weeds on crops. As rare species usually occur at moderate densities and have low competitive growth forms (e.g., small height and low biomass), it should be expected that yield losses are negligible. A thorough understanding of this relationship, however, is still missing.

To develop methods for implementing seed transfer as a practical tool to restore and increase agro‐biodiversity, this study aims to identify sowing rates which optimize the relationship between successful establishment of threatened species and minimal losses of crop yields. Therefore, a weed–crop competition experiment was established, including three rare arable species (*Legousia speculum‐veneris* (L.) Chaix, *Consolida regalis* Gray, *Lithospermum arvense* L.). The experiment was arranged as a partial additive design with increasing sowing rates in organically managed winter rye stands. Further more, the interaction among study species and the impact of spontaneous weeds were examined to answer the following questions:

1. How many seeds must be sown to obtain successful reintroduction of the study species?
2. What is the impact of increasing sowing rates on crop yield loss?

## Materials and Methods

### Study site

The experiment was set up on an organic farm in Gräfelfing, near Munich, Germany (543 m a.s.l., 48°07′ N, 11°25′ E). Soil texture of the study area was characterized by a high percentage of calcareous gravel which limits productivity due to low nutrient content and low water holding capacity (Fetzer et al. 1986). Means for annual temperature and precipitation were 7.9 °C and 953 mm (DWD 1996–2014). During the study period, the weather differed considerably from long‐term records. In autumn 2012, plant establishment was favoured by above‐average temperatures and lack of severe frost. This was followed by a long and cold winter, and a cool and rainy spring (DWD 2013). By mid‐June, summer started with a hot, sunny and dry phase which accelerated ripening of both crops and arable plants.

### Study species

We tested reintroduction of three rare arable weeds, that is, European Venus’ Looking Glass *Legousia speculum‐veneris* (L.) Chaix (Campanulaceae), Forking Larkspur *Consolida regalis* Gray (Ranunculaceae), and Field Gromwell *Lithospermum arvense* L. (Boraginaceae). They differ in seed size and weight with a thousand grain weight of 0.22–0.28, 0.98–1.80, and 3.0–6.0 g, respectively (Schneider et al. 1994). These winter annuals mainly occur in autumn‐sown cereals on moderately dry limestone soils (Kästner et al. 2001); they have limited dispersal and are often outcompeted by arable crops (Schneider et al. 1994). Although common 50–100 years ago, all three species are almost extinct from the study region today and are included in the Red List of Bavaria (Scheuerer and Ahlmer 2003). Autochthonous seeds were received from a local seed producer (Krimmer, Freising, Germany). Before sowing, germination was tested in a climate chamber for 6 weeks. Germination rates were 84% for *L. speculum‐veneris*, 73% for *C. regalis,* and 79% for *L. arvense*.

### Experimental design

At the beginning of October 2012, the winter rye (*Secale cereale*) variety “Danko” was sown in 40 plots with a plot seeder (Hans‐Ulrich Hege GmbH & Co. KG, Waldenburg, Germany) at 350 seeds m^−2^ and 11 cm row spacing. This resulted in 260 ± 4 eared stems m^−2^ (M. Lang, pers. observ., August 2013). Mean crop data in the region (variety “Danko”, year 2013, Bavaria) were stands with 372 ears m^−2^ and grain yields of 43.5 dt ha^−1^ (86% dry matter) (LfL 2013).

To study the effects of sowing rates on establishment and reproduction of arable plants as well as on crop yields, we chose a partial additive design (Keddy 2001). The plots measured 6.0 m × 1.1 m in size and were randomly arranged (Fig. S1). Seeds of the study species were applied to the plot centre at 3.0 m × 1.1 m immediately after sowing the crop. They were applied to the soil surface and rolled to improve soil contact and thus germination conditions. In 30 plots, each study species was sown individually (pure sowing) with ten sowing rates ranging from 5 to 10,000 seeds m^−2^ (Table 1). Additionally, ten plots were sown with a mixture of *L. arvense*,* C. regalis,* and *L. speculum‐veneris* at a ratio of 1:1.3:3.3 (mixed sowing). These proportions were chosen assuming lower establishment rates and minor competitiveness in small‐seeded species (cf. Moles and Westoby 2004). To achieve homogeneous distribution, seeds were mixed with 0.75 L soy meal per plot and sown by hand. This treatment led to a nutrient input of 70 kg N ha^−1^. During the growing season, no weed control measures or further fertilizers were applied.

**Table 1:** Reintroduction experiment with the rare arable plants *Legousia speculum‐veneris* (Leg), *Consolida regalis* (Con), and *Lithospermum arvense* (Lit) in pure and mixed sowing (ratio 1:1.3:3.3)

Pure sowing	Seeds (m^−2^)									
	5	10	25	50	100	200	500	1000	5000	10,000
Mixed sowing										
Leg	2.9	5.9	14.7	29.4	58.7	117.5	293.7	587.4	2937	5874
Con	1.2	2.3	5.8	11.6	23.1	46.3	115.7	231.4	1157	2314
Lit	0.9	1.8	4.5	8.9	17.8	35.6	89.0	178.0	890	1780
Total	5	10	25	50	100	200	500	1000	5000	10,000

Sampling was carried out in eight subplots per plot, each covering an area of 0.25 m² (77.0 cm × 23.5 cm) (Fig. S1): (1) Two subplots with sown study species and all other weeds regularly removed (starting in May 2013), (2) two subplots with sown study species and without weed removal, (3) two subplots without study species and weed removal, and (4) two subplots without study species and without weed removal.

Spontaneous weed vegetation included mainly common species, such as *Thlaspi arvense* L., *Stellaria media* agg., *Lamium purpureum* L.s.l., *Veronica persica* Poir., and *Lapsana communis* L.; highly competitive weeds, such as *Cirsium arvense* (L.) Scop. and *Galium aparine* L., were rare. Mean dry weight of spontaneous weed vegetation was 48 ± 2 g m^−2^ at harvest time (mean of subplots without weed removal).

### Establishment and reproductive success of study species, and crop yield

Establishment was measured as plant numbers of the study species before crop harvest in mid‐July 2013. Reproductive success was measured as seed production per area. This was achieved by multiplying mean seed numbers per fruit (counts of five fruits per species and subplot) with the number of fruits per fertile plant (counts of five individuals per species and subplot) and density of fertile plants (Kleyer et al. 2008). Establishment and reproduction were extrapolated to 1 m². To compare intra‐ and interspecific competition among the study species, the seed number in pure sowing was downscaled to the proportional seed number of each study species in mixed sowing (Table 1). Net reproduction rates (*Lambda*), which represent the rate of increase in annual plants (Silvertown and Charlesworth 2001), were calculated by dividing the number of seeds produced in summer 2013 by the number of seeds initially sown in autumn 2012.

To determine crop yield, rye plants of each subplot were manually harvested and rye ears were threshed in a stationary threshing machine. To standardize the results, grains were dried to 0% moisture and the weighted yield extrapolated to 86% dry matter and decitons per hectare.

### Statistical analyses

For statistical analyses, R Version 3.0.2 was used (R Core Team 2012). Response variables were calculated as mean numbers of two subplot samples per treatment. Sowing rates of 10,000 seeds m^−2^ for *C. regalis* were excluded from the analyses, due to feeding damages by roe deer in the respective plot.

The relationships between the response variables (establishment, seed production) and treatments, that is, sowing rates, type of sowing (pure vs. mixed sowing), weed removal (with vs. without removal) were calculated for each species separately using linear mixed‐effects models (lme) with a maximized log‐likelihood implemented in the *nlme* package (version 3.1‐111; Pinheiro et al. 2013). In this calculation, two‐way interactions of all explanatory parameters were considered and the variable “plot” was included as a random factor to model the independence of errors with respect to spatial autocorrelations. To achieve normal distribution of errors, sowing rates were log10‐transformed, seed production was square root‐transformed, and number of established individuals was log10(*x* + 1)‐transformed. Full models were simplified using an automatic backward stepwise selection procedure by AIC implemented in the *MASS* package (Version 7.3‐17; Ripley et al. 2013) until a minimal adequate model was obtained. Parameter estimates, *t*‐statistics, and *P*‐values of terms in the best model were taken from the summary table.

To identify the most appropriate method for characterizing the relationship between crop yield and sowing rates, hyperbolic (Cousens 1985), exponential (Poole and Gill 1987), and sigmoid (Williams and Hayes 1984) functions were tested with nonlinear least‐squares regressions (nls) using the *nlme* (Version 3.1‐111; Pinheiro et al. 2013) and *nlstools* (Version 0.0‐15; Baty and Delignette‐Muller 2013) packages. The sigmoid function fitted best:

$$y=a\cdot (1-b\cdot e^{-c\cdot x^{2}})$$

with *y *= crop yield [dt ha^−1^] and *x *= sowing rate of study species [m^−2^]. Parameters *a*,* b,* and *c* were estimated from regressions.

Each nonlinear regression was tested with grouped data for differences between the types of sowing (pure vs. mixed sowing) and weed removal (with vs. without). In case of nonsignificant differences between grouped data, simple nonlinear regressions were fitted. Both model functions were compared using an ANOVA table. In case of nonsignificant differences, the simpler model was chosen (Crawley 2002).

## Results

### Establishment

**Figure 1:**
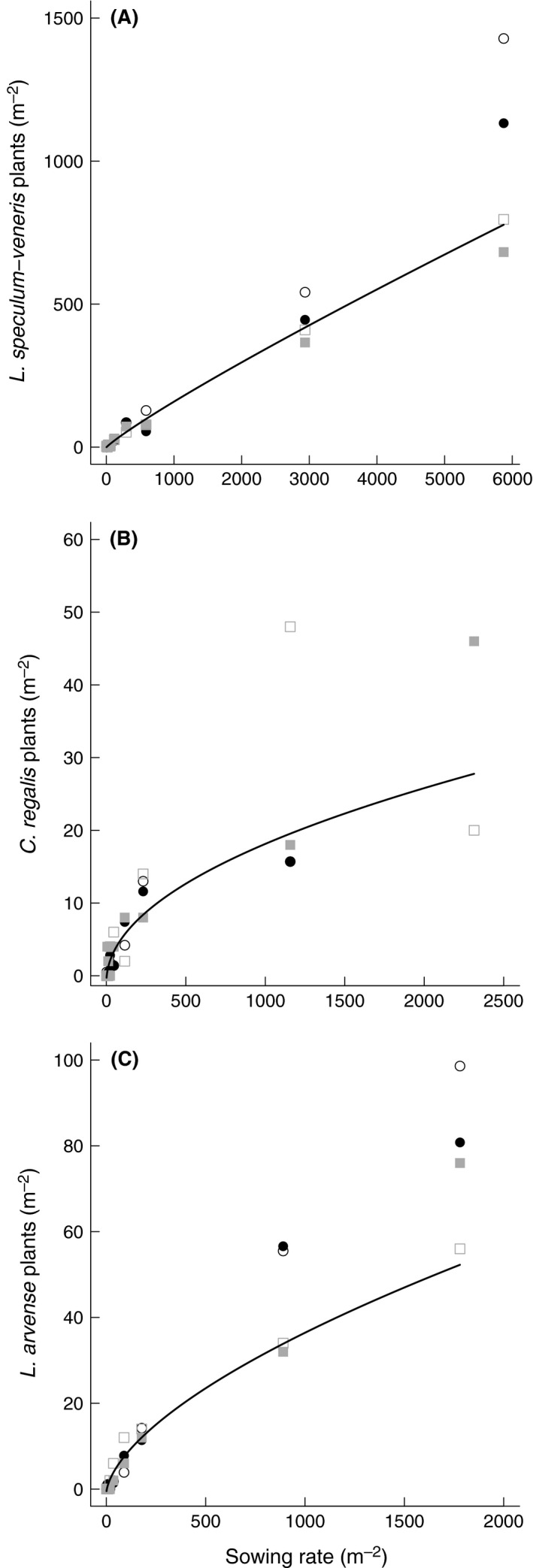
Establishment of the reintroduced rare arable plants *Legousia speculum‐veneris*,* Consolida regalis,* and *Lithospermum arvense* at harvest time in relation to sowing rate. Sowing of the arable plant species individually (circles), in mixture (quadrats), with and without removal of spontaneous weeds (unfilled and filled symbols). Lines indicate significant results for plant numbers (calculated with linear regression models); for better visibility, back‐transformed values are shown. Note the different scaling of axes.

All three study species established successfully from the transferred seeds. The mean establishment rate across sowing rates was 18.6 ± 2.4% for *L. speculum‐veneris*, 6.5 ± 2.1% for *C. regalis,* and 5.8 ± 1.2% for *L. arvense*. While establishment was low and patchy below sowing rates of 25 seeds m^−2^, an increase in sowing rates also increased the number of established plants (Table 2, Table S1). At very high sowing rates, saturated plant densities were approached for *C. regalis* and *L. arvense*, while this was not the case for *L. speculum‐veneris* (Fig. 1). Establishment was significantly affected by sowing rate, but not by sowing type (pure vs. mixed sowing of study species) and weed removal, as well as two‐way interaction between parameters (excluded from the minimal adequate model; results of the full models are shown in Table S2).

**Table 2:** Results of the minimal adequate Linear Mixed‐Effects Models for establishment and seed production of the three reintroduced arable plants at harvest time, with sowing rate as explanatory variable

	Value ± SE	df	*t*‐value	*P*‐value
**Establishment** a				
*Legousia speculum‐veneris*				
Intercept	−0.48 ± 0.10	20/20	−4.86	<0.001
Sowing rate^b^	0.89 ± 0.04	20/18	20.72	<0.001
*Consolida regalis*				
Intercept	−0.18 ± 0.07	19/19	−2.71	0.014
Sowing rate^c^	0.49 ± 0.04	19/17	13.50	<0.001
*Lithospermum arvense*				
Intercept	−0.26 ± 0.08	20/20	−3.33	0.003
Sowing rate^c^	0.61 ± 0.04	20/18	14.23	<0.001
**Seed production** b				
*Legousia speculum‐veneris*				
Intercept	−74.06 ± 18.38	20/20	−4.03	<0.001
Sowing rate^c^	132.33 ± 8.08	20/18	16.37	<0.001
*Consolida regalis*				
Intercept	−1.12 ± 4.54	20/19	−0.25	0.808
Sowing rate^c^	15.89 ± 2.50	20/17	6.37	<0.001
*Lithospermum arvense*				
Intercept	−11.28 ± 3.99	20/20	−2.83	0.010
Sowing rate^c^	23.70 ± 2.18	20/18	10.87	<0.001

a log10(*x* + 1)‐transformed.

b Square root‐transformed.

c log10‐transformed.

### Seed production

**Figure 2:**
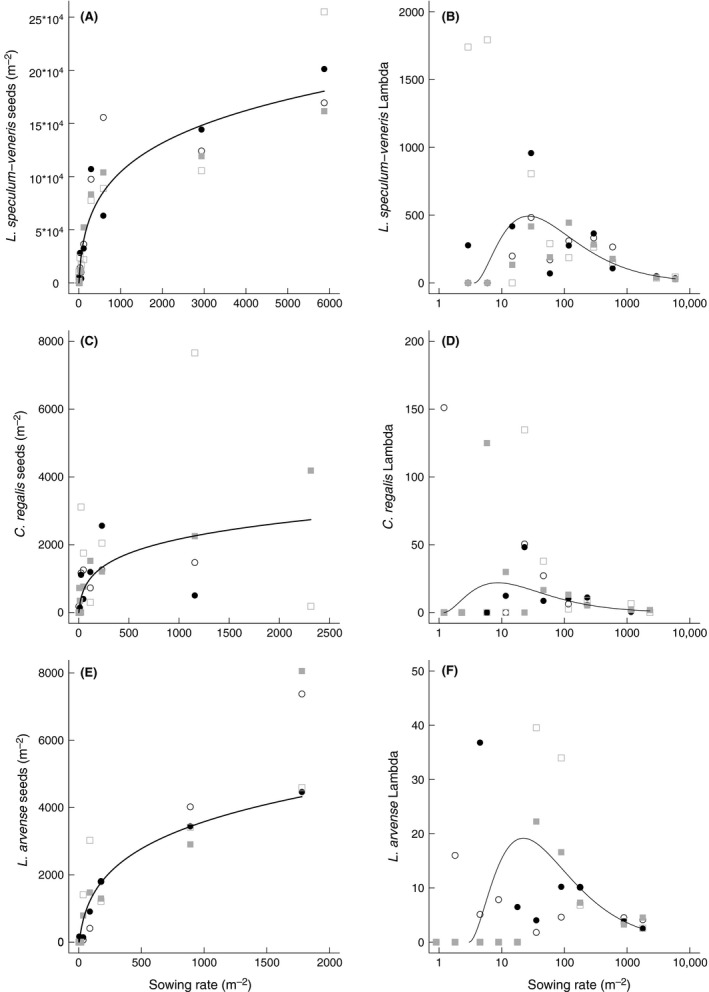
Seed production (left) and Lambda (right) of the reintroduced rare arable plants *Legousia speculum‐veneris*,* Consolida regalis,* and *Lithospermum arvense* in relation to sowing rate. Sowing of study species: individually (circles), in species mixtures (quadrats), with and without removal of spontaneous weeds (unfilled and filled symbols). Lines indicate significant results for seed production and Lambda (calculated with linear regression models). For better visibility, back‐transformed values are shown; note the different scalings of axes.

Seed production was highest for *L. speculum‐veneris* (255,000 seeds m^−2^), followed by *L. arvense* (8100 seeds m^−2^) and *C. regalis* (4200 seeds m^−2^) (Table S1). In most experimental plots, seed production of the study species exceeded the number of initially sown seeds. Only at low sowing rates (<25 seeds m^−2^), seed production was occasionally inhibited due to the lack of established plants (Fig. 2, left column; Table S1). Seed production significantly increased with the number of sown seeds for all three study species (Table 2). In comparison to plant numbers, however, seed production strongly leveled off at higher sowing rates. There was no influence of sowing type (pure vs. mixed sowing of study species) and weed removal on seed production of all study species. Two‐way interaction could also be excluded from the minimal adequate model (Table S2).

Numbers of seeds per surviving plant strongly differed between sowing rates and tended to decrease with increasing sowing rate (Table S1). For *L. speculum‐veneris,* the minimum value was 119 seeds per plant (pure sowing at 10,000 seeds m^−2^, weed removal). The maximum value was 50 times higher with 6035 seeds per plant (pure sowing at 50 seeds m^−2^, weed removal). In *C. regalis,* the number of seeds per plant varied from 3 (pure sowing at 10,000 seeds m^−2^, no weed removal) to 905 (pure sowing at 200 seeds m^−2^, weed removal). These differences were less pronounced in *L. arvense* with a minimum number of seeds per plant of 36 (mixed sowing at 35.6 seeds m^−2^, weed removal) and a maximum of 396 (pure sowing at 200 seeds m^−2^, no weed removal).

As Lambda reflected the ratio of seed input to output, the peak of the Lambda curve indicated the sowing rate with highest seed production per sown seed (Fig. 2, right column). Highest Lambda was predicted for *L. speculum‐veneris* at a sowing rate of 27 seeds m^−2^ (Λ = 493), for *C. regalis* at 22 seeds m^−2^ (Λ = 9), and for *L. arvense* at 19 seeds m^−2^ (Λ = 22). Above those sowing rates, Lambda values declined again.

### Crop yield

**Figure 3:**
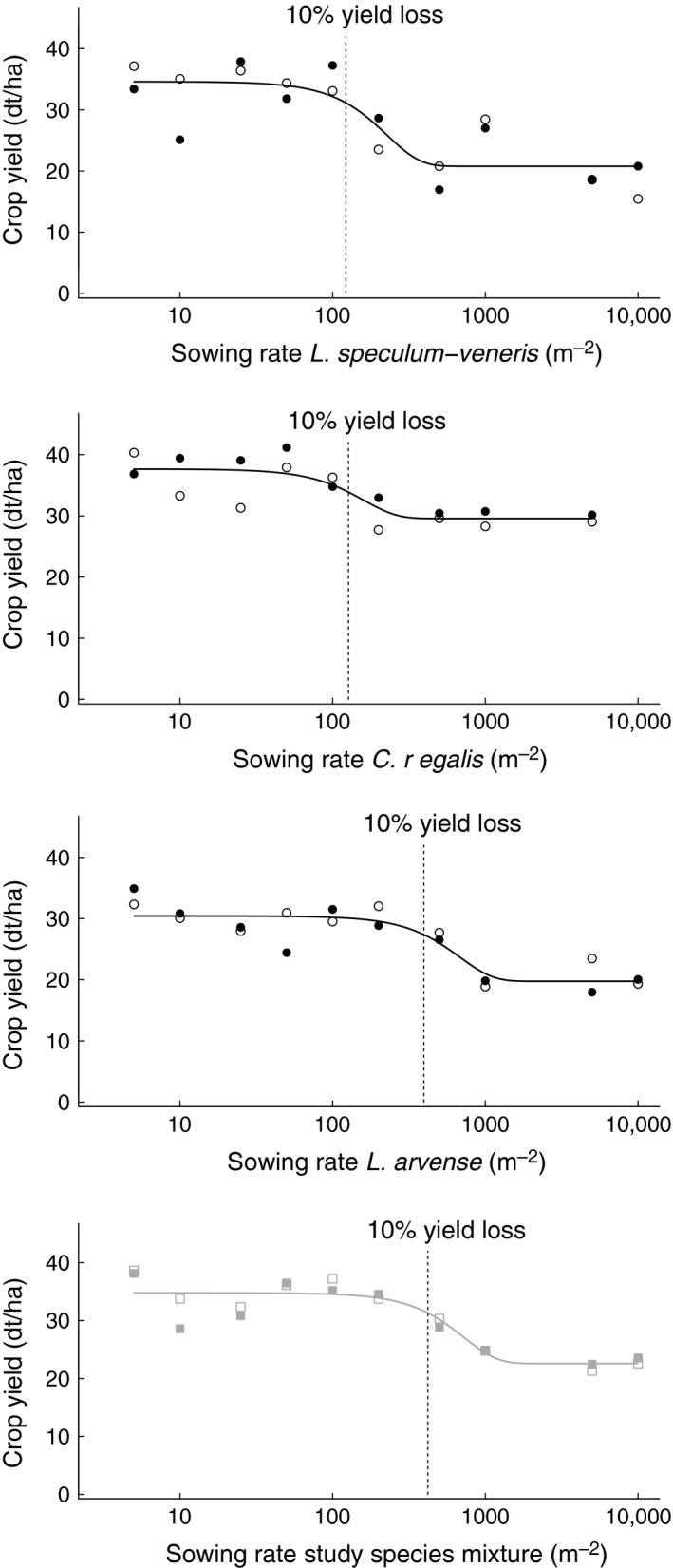
Relationship between rye yield (86% dry matter) and sowing rate of the three reintroduced arable plants. Sowing of the rare species individually (circles), in species mixtures (quadrats), with and without removal of spontaneous weeds (unfilled and filled symbols). Lines indicate significant results for crop yield (calculated with nonlinear regression models by Williams and Hayes 1984), the *x*‐axis is log‐scaled, and dotted lines indicate 10% yield loss.

The mean crop yield was 31.2 ± 3.2 dt ha^−1^ without study species. With study species, there was no effect of weed removal on crop yield (Table 3, Fig. 3). Accordingly, almost no yield losses occurred below sowing rates of 100 seeds m^−2^ for all study species. With increasing sowing rates, a clear decline in crop yield was observed. However, at more than 1000 seeds m^−2^, stable yields were regained. An economically relevant yield loss of 10% occurred at 120 seeds m^−2^ for *L. speculum‐veneris*, at 130 seeds m^−2^ for *C. regalis*, and at 400 seeds m^−2^ for *L. arvense*. Sowing a mixture of study species led to 10% yield loss at a sowing rate of 410 seeds m^−2^.

**Table 3:** Parameters of the sigmoid model $y=a\cdot (1-b\cdot e^{-c\cdot x^{2}})$ describing crop yield of rye as a function of sowing rates of three reintroduced arable plants sown individually and in mixture

Parameter	Estimate ± SE	df	*t*‐value	*P*‐value
*Legousia speculum–veneris*				
a	20.78 ± 1.54	17	13.50	<0.001
b	−0.67 ± 0.14	17	−4.71	<0.001
c	1.93 × 10^−5^ ± 1.23 × 10^−5^	17	1.57	0.135
*Consolida regalis*				
a	29.59 ± 1.10	15	26.89	<0.001
b	−0.27 ± 0.06	15	−4.79	<0.001
c	3.90 × 10^−5^ ± 2.59 × 10^−5^	15	1.51	0.152
*Lithospermum arvense*				
a	19.76 ± 1.18	17	16.70	<0.001
b	−0.54 ± 0.10	17	−5.55	<0.001
c	2.15 × 10^−6^ ± 1.04 × 10^−6^	17	2.06	0.055
Species mixture				
a	22.58 ± 1.20	17	18.75	<0.001
b	−0.54 ± 0.09	17	−6.23	<0.001
c	1.89 × 10^−6^ ± 7.94 × 10^−7^	17	2.39	0.029

## Discussion

### Species‐specific establishment

All three study species established successfully in nearly all treatments of the field experiment. Successful establishment of a reintroduced species is achieved when the number of seeds produced by the study species exceeds the number of seeds used for initial sowing (Pavlik 1996). If this net reproduction rate is greater than one, the population grows (Silvertown and Charlesworth 2001). As random demographic events or environmental fluctuations particularly threaten small populations, high reproduction rates in the initial phase of restoration are desirable to rapidly reduce these risks and to sustainably ensure survival. In our study, sowing rates above 20 seeds m^−2^ resulted in Lambda values above three (Table S1). Thus, for endangered winter annuals, we could show that rare arable species are suited for the transfer to fields with favourable establishment conditions. This was also proven by the study of Mayer et al. (2012). They studied seven rare arable species and found varying establishment rates, reaching from 0.6% in *C. regalis* to 7.2% in *Melampyrum arvense,* 1 year after sowing. Early sowing, no weed control, no under‐sown crops (Albrecht et al. 2009), and suitable weather conditions during the establishment phase of study species (Rühl et al. 2015) may have favoured establishment success in our study.

Usually, seedlings of large‐seeded taxa show more successful establishment and higher survival rates than small‐seeded species (Moles and Westoby 2004). In the present study, however, small‐seeded *L. speculum‐veneris* developed better than the two large‐seeded species. Species‐specific temperature requirements for germination could be a reason for these differences. Seeds of *L. arvense* and *C. regalis* sampled from the Munich plain showed strong preference for low germination temperatures, while *L. speculum‐veneris* germinated at a much broader spectrum of temperatures (Otte 1996). Warm temperatures in autumn may have favoured germination of *L. speculum‐veneris* in our study that was performed for only 1 year. Arable plant populations fluctuate with annual weather conditions (McCloskey et al. 1996). Thus, studies over several years are needed to investigate the species‐specific establishment success. However, as literature about reestablishment of rare arable plant species is scarce up to now, our study provides first and valuable insights for reintroducing plant diversity to arable landscapes.

### Density effects

The relationship between sowing rates and plant numbers was shaped by density effects, especially in *C. regalis* and *L. arvense*. This can be attributed to reduced germination (Palmblad 1968; Bergelson and Perry 1989; Murray 1998) or increased mortality (Morris 2003; Weiner and Freckleton 2010) at high sowing rates. Mortality may not only be restricted to resource limitation, because at high sowing rates, numerous plants of *C. regalis* dried up due to fungal infections (M. Lang, pers. observ.). This observation confirms that dense plant populations are susceptible to fungal diseases or herbivorous parasites (Burdon and Chilvers 1975).

All three study species showed strong density effects for seed production, which reflects the impact of phenotypic plasticity in addition to density‐dependent germination and mortality (Silvertown and Charlesworth 2001). In particular, in the case of *L. speculum‐veneris,* size and reproduction per survivor were significantly reduced with increasing sowing rate. In accordance with the ecological principle of constant final yield (Weiner and Freckleton 2010), seed production per area was increasingly saturated at very high sowing rates. However, the limit of saturation was not reached. A plastic response in size and reproductive capacity to increasing density was shown for several annual weed species, for example, *Agrostemma githago* (Harper and Gajic 1961) or *Papaver* spec. (Harper and McNaughton 1962). In populations of autogamous species, high numbers of individuals with a low number of seeds can more efficiently maintain genetic variation than few individuals with many seeds (Harper and Gajic 1961). In the latter case, mortality would strongly reduce genetic variation.

There was no significant difference in establishment and seed production between sowing the study species individually or in mixture. Usually, intraspecific competition is stronger than interspecific competition, as individual plants compete for the same resources and are equally threatened by predators and diseases (Amarasekare 2003). These interactions, however, strongly depend on the particular species and the composition of the plant community. As weak competitors (compared to noxious weeds such as *Galium aparine* L.), our study species could even have benefited from intraspecific aggregation (Stoll and Prati 2001). Furthermore, our study species have some traits in common (e.g. plant life cycle of winter annuals) and similar resource requirements for relatively warm and sunny sites (Kleyer et al. 2008), which led to similar intra‐ and interspecific competition. However, none of the three study species was outcompeted at high sowing rates, which can be attributed to their generally low competitiveness.

Competition with the spontaneous weed vegetation had no influence on the establishment and seed production of the study species. This may be due to a lack of highly competitive weeds in the study plots. Testing for competition in a mixture of *L. speculum‐veneris*,* Stellaria media* and wheat, Epperlein et al. (2014) found no significant impact of *S. media* on *L. speculum‐veneris*. As *S. media* only has an intermediate competitive ability (Marshall et al. 2003), the authors conclude that the most important factor for seed or biomass production in *L. speculum‐veneris* is the strength of the strongest competitor.

### Impact on crop yield

Hyperbolic models are often used to describe crop yield losses related to weed density or weed biomass (Zimdahl 2004). In our study, very low sowing rates resulted in only few plants with comparatively low competitive ability. Consequently, no effect on crop yield could be measured up to sowing rates of 100 seeds m^−2^. At very high sowing rates (>1000 seeds m^−2^), competition among the study species reduced their competitive ability toward the crop and no further yield losses were recorded. Thus, the relationship between sowing rates of rare arable plants and crop yield could be described best by sigmoid models. Compared to the competitiveness of different annual weed species toward crop yield in Wilson and Wright (1990), our study species were not as weak competitors as expected. This can be attributed to a very low crop density (Bayerische Landesanstalt für Landwirtschaft (LfL) 2013) which generally enhances competition effects by weeds (Wilson et al. 1995). Fields with low nutrient content and comparatively low crop density occur in several parts of Europe, especially on organic farms. Such sites should be chosen when reintroducing rare arable plants. In nutrient‐rich arable fields, we expect a higher competitiveness of rye and lower crop yield losses caused by the rare arable plants (Rotchés‐Ribalta et al. 2015b).

### Synthesis and applications

This study shows that reintroduction of rare arable plants depends on appropriate sowing rates. Sowing rates <25 seeds m^−2^ should be avoided as seedling establishment is very uncertain, while rates of >1000 seeds m^−2^ cause negative density effects and reduce crop yields. Balancing crop yield losses (<10%) and optimum reintroduction success of the study species, we recommend mixtures with 50 seeds m^−2^ for *L. speculum‐veneris* and 100 seeds m^−2^ for both *C. regalis* and *L. arvense*. As Lambda usually exceeded the value of 1.0 in our study, these sowing rates should produce successful establishment under suitable growing conditions. In small‐sized study areas, sowing rates should be increased to maintain an adequate genetic diversity of introduced populations (Bischoff et al. 2010). Seed production of rare arable plants is one of the highest cost factors in reintroduction measures (M. Lang, unpubl. results). Therefore, management operations in the year of transfer should focus on successful establishment of the threatened species and waive weed control or cultivation of highly competitive crops. In organic farming, no harrowing and reduced sowing rates of the crop would benefit the establishment success of rare arable plants. Establishment conditions should be orientated to the specific requirements of expensive species such as *L. arvense*. Considering this advice, seed transfer to suitable sites could enhance agro‐biodiversity and improve the ecosystem services of arable fields without generating inacceptable yield losses and recurring costs for reseeding.

## Conflict of Interest

None declared.

## Supporting information


**Table S1.** Establishment and reproduction of the study species *Legousia speculum‐veneris* (Leg), *Consolida regalis* (Con) and *Lithospermum arvense* (Lit) in pure and mixed sowing, with and without removal of spontaneous weeds.
**Table S2.** Results of the full Linear Mixed‐Effects Models for establishment and seed production of the three re‐introduced arable plants at harvest time, with sowing rate, sowing type (pure and mixed sowing), weed removal (with and without removal of spontaneous weeds), and all two‐way interactions as explanatory variables.
**Figure S1.** Schematic illustration of the partial additive study design.
