# Supplementary material for: Reintroduction of rare arable plants by seed transfer. What are the optimal sowing rates?
Source: Ecol Evol. 2016 Jul 12;6(15):5506–16. doi: 10.1002/ece3.2303 (PMC4984521; doi:10.1002/ece3.2303)

**Supplementary information**

**Table S1.** Establishment and reproduction of the study species *Legousia speculum-veneris* (Leg), *Consolida regalis* (Con) and *Lithospermum arvense* (Lit) in pure and mixed sowing, with and without removal of spontaneous weeds. To compare intra- and interspecific competition among the study species, downscaled numbers of plants and seed production were calculated. This was done for pure sowing by referring to the proportional number of sown seeds of each study species in mixed sowing.

| Study species | Sowing type | Removal of spontaneous weeds | Sown seeds [m^-2^] | Plants [m^-2^] | Seed production [m^-2^] | Seeds per plant | Lambda | Downscaled number of sown seeds [m^-2^] | Downscaled number of plants [m^-2^] | Downscaled number of seed production [m^-2^] |
| --- | --- | --- | --- | --- | --- | --- | --- | --- | --- | --- |
| Leg | pure | no removal | 5 | 2 | 1386 | 693 | 277 | 2.9 | 1.2 | 804 |
| Leg | pure | no removal | 10 | 0 | 0 | na | 0 | 5.9 | 0.0 | 0 |
| Leg | pure | no removal | 25 | 8 | 10436 | 1305 | 417 | 14.7 | 4.7 | 6137 |
| Leg | pure | no removal | 50 | 12 | 47886 | 3990 | 958 | 29.4 | 7.1 | 28157 |
| Leg | pure | no removal | 100 | 10 | 6975 | 698 | 70 | 58.7 | 5.9 | 4094 |
| Leg | pure | no removal | 200 | 46 | 55122 | 1198 | 276 | 117.5 | 27.0 | 32384 |
| Leg | pure | no removal | 500 | 146 | 182278 | 1248 | 365 | 293.7 | 85.8 | 107070 |
| Leg | pure | no removal | 1000 | 94 | 107630 | 1145 | 108 | 587.4 | 55.2 | 63222 |
| Leg | pure | no removal | 5000 | 758 | 245584 | 324 | 49 | 2937.0 | 445.2 | 144256 |
| Leg | pure | no removal | 10000 | 1928 | 342528 | 178 | 34 | 5874.0 | 1132.5 | 201201 |
| Con | pure | no removal | 5 | 0 | 0 | na | 0 | 1.2 | 0.0 | 0 |
| Con | pure | no removal | 10 | 0 | 0 | na | 0 | 2.3 | 0.0 | 0 |
| Con | pure | no removal | 25 | 0 | 0 | na | 0 | 5.8 | 0.0 | 0 |
| Con | pure | no removal | 50 | 2 | 616 | 308 | 12 | 11.6 | 0.5 | 143 |
| Con | pure | no removal | 100 | 12 | 4822 | 402 | 48 | 23.1 | 2.8 | 1114 |
| Con | pure | no removal | 200 | 6 | 1723 | 287 | 9 | 46.3 | 1.4 | 399 |
| Con | pure | no removal | 500 | 32 | 5171 | 162 | 10 | 115.7 | 7.4 | 1197 |
| Con | pure | no removal | 1000 | 50 | 11070 | 221 | 11 | 231.4 | 11.6 | 2562 |
| Con | pure | no removal | 5000 | 68 | 2176 | 32 | 0 | 1157.0 | 15.7 | 504 |
| Con | pure | no removal | 10000 | 6 | 16 | 3 | 0 | 2314.0 | 1.4 | 4 |
| Lit | pure | no removal | 5 | 0 | 0 | na | 0 | 0.9 | 0.0 | 0 |
| Lit | pure | no removal | 10 | 0 | 0 | na | 0 | 1.8 | 0.0 | 0 |
| Lit | pure | no removal | 25 | 6 | 920 | 153 | 37 | 4.5 | 1.1 | 166 |
| Lit | pure | no removal | 50 | 0 | 0 | na | 0 | 8.9 | 0.0 | 0 |
| Lit | pure | no removal | 100 | 6 | 648 | 108 | 6 | 17.8 | 1.1 | 115 |
| Lit | pure | no removal | 200 | 8 | 806 | 101 | 4 | 35.6 | 1.4 | 144 |
| Lit | pure | no removal | 500 | 44 | 5104 | 116 | 10 | 89.0 | 7.8 | 909 |
| Lit | pure | no removal | 1000 | 64 | 10189 | 159 | 10 | 178.0 | 11.4 | 1814 |
| Lit | pure | no removal | 5000 | 318 | 19334 | 61 | 4 | 890.0 | 56.6 | 3442 |
| Lit | pure | no removal | 10000 | 454 | 25061 | 55 | 3 | 1780.0 | 80.8 | 4461 |
| Leg | pure | removal | 5 | 0 | 0 | na | 0 | 2.9 | 0.0 | 0 |
| Leg | pure | removal | 10 | 0 | 0 | na | 0 | 5.9 | 0.0 | 0 |
| Leg | pure | removal | 25 | 6 | 4949 | 825 | 198 | 14.7 | 3.5 | 2910 |
| Leg | pure | removal | 50 | 4 | 24142 | 6035 | 483 | 29.4 | 2.4 | 14195 |
| Leg | pure | removal | 100 | 8 | 17072 | 2134 | 171 | 58.7 | 4.7 | 10021 |
| Leg | pure | removal | 200 | 40 | 61988 | 1550 | 310 | 117.5 | 23.5 | 36418 |
| Leg | pure | removal | 500 | 146 | 166060 | 1137 | 332 | 293.7 | 85.8 | 97544 |
| Leg | pure | removal | 1000 | 218 | 265012 | 1216 | 265 | 587.4 | 128.1 | 155668 |
| Leg | pure | removal | 5000 | 922 | 211322 | 229 | 42 | 2937.0 | 541.6 | 124131 |
| Leg | pure | removal | 10000 | 2432 | 288362 | 119 | 29 | 5874.0 | 1428.6 | 169384 |
| Con | pure | removal | 5 | 2 | 756 | 378 | 151 | 1.2 | 0.5 | 181 |
| Con | pure | removal | 10 | 0 | 0 | na | 0 | 2.3 | 0.0 | 0 |
| Con | pure | removal | 25 | 0 | 0 | na | 0 | 5.8 | 0.0 | 0 |
| Con | pure | removal | 50 | 0 | 0 | na | 0 | 11.6 | 0.0 | 0 |
| Con | pure | removal | 100 | 8 | 5055 | 632 | 51 | 23.1 | 1.8 | 1168 |
| Con | pure | removal | 200 | 6 | 5429 | 905 | 27 | 46.3 | 1.4 | 1257 |
| Con | pure | removal | 500 | 18 | 3144 | 175 | 6 | 115.7 | 4.2 | 727 |
| Con | pure | removal | 1000 | 56 | 5443 | 97 | 5 | 231.4 | 13.0 | 1260 |
| Con | pure | removal | 5000 | 68 | 6378 | 94 | 1 | 1157.0 | 15.7 | 1476 |
| Con | pure | removal | 10000 | 0 | 0 | na | 0 | 2314.0 | 0.0 | 0 |
| Lit | pure | removal | 5 | 0 | 0 | na | 0 | 0.9 | 0.0 | 0 |
| Lit | pure | removal | 10 | 4 | 160 | 40 | 16 | 1.8 | 0.7 | 29 |
| Lit | pure | removal | 25 | 2 | 128 | 64 | 5 | 4.5 | 0.4 | 23 |
| Lit | pure | removal | 50 | 6 | 392 | 65 | 8 | 8.9 | 1.1 | 70 |
| Lit | pure | removal | 100 | 0 | 0 | 0 | 0 | 17.8 | 0.0 | 0 |
| Lit | pure | removal | 200 | 10 | 360 | 36 | 2 | 35.6 | 1.8 | 64 |
| Lit | pure | removal | 500 | 22 | 2306 | 105 | 5 | 89.0 | 3.9 | 410 |
| Lit | pure | removal | 1000 | 80 | 10112 | 126 | 10 | 178.0 | 14.2 | 1800 |
| Lit | pure | removal | 5000 | 312 | 22589 | 72 | 5 | 890.0 | 55.5 | 4021 |
| Lit | pure | removal | 10000 | 554 | 41439 | 75 | 4 | 1780.0 | 98.6 | 7376 |
| Leg | mixed | no removal | 2.9 | 0 | 0 | na | 0 | 2.9 | 0.0 | 0 |
| Leg | mixed | no removal | 5.9 | 0 | 0 | na | 0 | 5.9 | 0.0 | 0 |
| Leg | mixed | no removal | 14.7 | 2 | 1958 | 979 | 133 | 14.7 | 2.0 | 1958 |
| Leg | mixed | no removal | 29.4 | 8 | 12251 | 1531 | 417 | 29.4 | 8.0 | 12251 |
| Leg | mixed | no removal | 58.7 | 2 | 11118 | 5559 | 189 | 58.7 | 2.0 | 11118 |
| Leg | mixed | no removal | 117.5 | 30 | 52229 | 1741 | 445 | 117.5 | 30.0 | 52229 |
| Leg | mixed | no removal | 293.7 | 72 | 83246 | 1156 | 283 | 293.7 | 72.0 | 83246 |
| Leg | mixed | no removal | 587.4 | 80 | 103936 | 1299 | 177 | 587.4 | 80.0 | 103936 |
| Leg | mixed | no removal | 2937.0 | 366 | 119199 | 326 | 41 | 2937.0 | 366.0 | 119199 |
| Leg | mixed | no removal | 5874.0 | 682 | 161525 | 237 | 27 | 5874.0 | 682.0 | 161525 |
| Con | mixed | no removal | 1.2 | 0 | 0 | na | 0 | 1.2 | 0.0 | 0 |
| Con | mixed | no removal | 2.3 | 0 | 0 | na | 0 | 2.3 | 0.0 | 0 |
| Con | mixed | no removal | 5.8 | 4 | 725 | 181 | 125 | 5.8 | 4.0 | 725 |
| Con | mixed | no removal | 11.6 | 2 | 347 | 174 | 30 | 11.6 | 2.0 | 347 |
| Con | mixed | no removal | 23.1 | 0 | 0 | na | 0 | 23.1 | 0.0 | 0 |
| Con | mixed | no removal | 46.3 | 4 | 764 | 191 | 17 | 46.3 | 4.0 | 764 |
| Con | mixed | no removal | 115.7 | 8 | 1525 | 191 | 13 | 115.7 | 8.0 | 1525 |
| Con | mixed | no removal | 231.4 | 8 | 1210 | 151 | 5 | 231.4 | 8.0 | 1210 |
| Con | mixed | no removal | 1157.0 | 18 | 2253 | 125 | 2 | 1157.0 | 18.0 | 2253 |
| Con | mixed | no removal | 2314.0 | 46 | 4190 | 91 | 2 | 2314.0 | 46.0 | 4190 |
| Lit | mixed | no removal | 0.9 | 0 | 0 | na | 0 | 0.9 | 0.0 | 0 |
| Lit | mixed | no removal | 1.8 | 0 | 0 | na | 0 | 1.8 | 0.0 | 0 |
| Lit | mixed | no removal | 4.5 | 0 | 0 | na | 0 | 4.5 | 0.0 | 0 |
| Lit | mixed | no removal | 8.9 | 0 | 0 | na | 0 | 8.9 | 0.0 | 0 |
| Lit | mixed | no removal | 17.8 | 0 | 0 | na | 0 | 17.8 | 0.0 | 0 |
| Lit | mixed | no removal | 35.6 | 2 | 792 | 396 | 22 | 35.6 | 2.0 | 792 |
| Lit | mixed | no removal | 89.0 | 6 | 1476 | 246 | 17 | 89.0 | 6.0 | 1476 |
| Lit | mixed | no removal | 178.0 | 12 | 1296 | 108 | 7 | 178.0 | 12.0 | 1296 |
| Lit | mixed | no removal | 890.0 | 32 | 2906 | 91 | 3 | 890.0 | 32.0 | 2906 |
| Lit | mixed | no removal | 1780.0 | 76 | 8056 | 106 | 5 | 1780.0 | 76.0 | 8056 |
| Leg | mixed | removal | 2.9 | 2 | 5046 | 2523 | 1740 | 2.9 | 2.0 | 5046 |
| Leg | mixed | removal | 5.9 | 4 | 10582 | 2646 | 1794 | 5.9 | 4.0 | 10582 |
| Leg | mixed | removal | 14.7 | 0 | 0 | na | 0 | 14.7 | 0.0 | 0 |
| Leg | mixed | removal | 29.4 | 8 | 23694 | 2962 | 806 | 29.4 | 8.0 | 23694 |
| Leg | mixed | removal | 58.7 | 6 | 17035 | 2839 | 290 | 58.7 | 6.0 | 17035 |
| Leg | mixed | removal | 117.5 | 26 | 21970 | 845 | 187 | 117.5 | 26.0 | 21970 |
| Leg | mixed | removal | 293.7 | 52 | 77571 | 1492 | 264 | 293.7 | 52.0 | 77571 |
| Leg | mixed | removal | 587.4 | 78 | 88798 | 1138 | 151 | 587.4 | 78.0 | 88798 |
| Leg | mixed | removal | 2937.0 | 410 | 105534 | 257 | 36 | 2937.0 | 410.0 | 105534 |
| Leg | mixed | removal | 5874.0 | 796 | 255022 | 320 | 43 | 5874.0 | 796.0 | 255022 |
| Con | mixed | removal | 1.2 | 0 | 0 | na | 0 | 1.2 | 0.0 | 0 |
| Con | mixed | removal | 2.3 | 0 | 0 | na | 0 | 2.3 | 0.0 | 0 |
| Con | mixed | removal | 5.8 | 0 | 0 | na | 0 | 5.8 | 0.0 | 0 |
| Con | mixed | removal | 11.6 | 0 | 0 | na | 0 | 11.6 | 0.0 | 0 |
| Con | mixed | removal | 23.1 | 4 | 3113 | 778 | 135 | 23.1 | 4.0 | 3113 |
| Con | mixed | removal | 46.3 | 6 | 1754 | 292 | 38 | 46.3 | 6.0 | 1754 |
| Con | mixed | removal | 115.7 | 2 | 299 | 149 | 3 | 115.7 | 2.0 | 299 |
| Con | mixed | removal | 231.4 | 14 | 2047 | 146 | 9 | 231.4 | 14.0 | 2047 |
| Con | mixed | removal | 1157.0 | 48 | 7661 | 160 | 7 | 1157.0 | 48.0 | 7661 |
| Con | mixed | removal | 2314.0 | 20 | 184 | 9 | 0 | 2314.0 | 20.0 | 184 |
| Lit | mixed | removal | 0.9 | 0 | 0 | na | 0 | 0.9 | 0.0 | 0 |
| Lit | mixed | removal | 1.8 | 0 | 0 | na | 0 | 1.8 | 0.0 | 0 |
| Lit | mixed | removal | 4.5 | 0 | 0 | na | 0 | 4.5 | 0.0 | 0 |
| Lit | mixed | removal | 8.9 | 0 | 0 | na | 0 | 8.9 | 0.0 | 0 |
| Lit | mixed | removal | 17.8 | 2 | 0 | 0 | 0 | 17.8 | 2.0 | 0 |
| Lit | mixed | removal | 35.6 | 6 | 1408 | 235 | 40 | 35.6 | 6.0 | 1408 |
| Lit | mixed | removal | 89.0 | 12 | 3024 | 252 | 34 | 89.0 | 12.0 | 3024 |
| Lit | mixed | removal | 178.0 | 14 | 1213 | 87 | 7 | 178.0 | 14.0 | 1213 |
| Lit | mixed | removal | 890.0 | 34 | 3414 | 100 | 4 | 890.0 | 34.0 | 3414 |
| Lit | mixed | removal | 1780.0 | 56 | 4592 | 82 | 3 | 1780.0 | 56.0 | 4592 |

**Table S2.** Results of the full Linear Mixed-Effects Models for establishment and seed production of the three re-introduced arable plants at harvest time, with sowing rate, sowing type (pure and mixed sowing), weed removal (with and without removal of spontaneous weeds), and all two-way interactions as explanatory variables.

|  | Value ± SE | df | t-value | p-value |
| --- | --- | --- | --- | --- |
| **Establishment*** |  |  |  |  |
| *Legousia speculum-veneris* |  |  |  |  |
| Intercept | –0.51 ± 0.16 | 20/17 | –3.07 | 0.007 |
| Sowing rate‡ | 0.93 ± 0.07 | 20/16 | 13.11 | <0.001 |
| Mixed sowing¶ | 0.05 ± 0.21 | 20/16 | 0.24 | 0.815 |
| With weed removal§ | –0.06 ± 0.16 | 20/17 | –0.40 | 0.694 |
| Sowing rate : Mixed sowing¶ | –0.08 ± 0.09 | 20/16 | –0.90 | 0.384 |
| Sowing rate : With weed removal§ | 0.01 ± 0.06 | 20/17 | 0.17 | 0.869 |
| Mixed sowing¶ : With weed removal§ | 0.14 ± 0.13 | 20/17 | 1.06 | 0.305 |
| *Consolida regalis* |  |  |  |  |
| Intercept | –0.16 ± 0.13 | 19/16 | –1.26 | 0.224 |
| Sowing rate‡ | 0.46 ± 0.07 | 19/15 | 6.46 | <0.001 |
| Mixed sowing¶ | 0.05 ± 0.16 | 19/15 | 0.32 | 0.751 |
| With weed removal§ | –0.06 ± 0.15 | 19/16 | –0.40 | 0.695 |
| Sowing rate : Mixed sowing¶ | 0.03 ± 0.08 | 19/15 | 0.38 | 0.711 |
| Sowing rate : With weed removal§ | 0.02 ± 0.08 | 19/16 | 0.26 | 0.796 |
| Mixed sowing¶ : With weed removal§ | –0.02 ± 0.15 | 19/16 | –0.16 | 0.878 |
| *Lithospermum arvense* |  |  |  |  |
| Intercept | –0.26 ± 0.13 | 20/17 | –2.02 | 0.059 |
| Sowing rate‡ | 0.62 ± 0.07 | 20/16 | 9.04 | <0.001 |
| Mixed sowing¶ | –0.08 ± 0.17 | 20/16 | –0.47 | 0.647 |
| With weed removal§ | 0.02 ± 0.09 | 20/17 | 0.23 | 0.821 |
| Sowing rate : Mixed sowing¶ | –0.01 ± 0.09 | 20/16 | –0.06 | 0.954 |
| Sowing rate : With weed removal§ | –0.01 ± 0.04 | 20/17 | –0.27 | 0.787 |
| Mixed sowing¶ : With weed removal§ | 0.10 ± 0.09 | 20/17 | 1.12 | 0.280 |
| **Seed production**† |  |  |  |  |
| *Legousia speculum-veneris* |  |  |  |  |
| Intercept | –84.29 ± 32.27 | 20/17 | –2.61 | 0.018 |
| Sowing rate‡ | 137.17 ± 13.86 | 20/16 | 9.90 | <0.001 |
| Mixed sowing¶ | 9.12 ± 41.55 | 20/16 | 0.22 | 0.829 |
| With weed removal§ | 3.50 ± 33.12 | 20/17 | 0.11 | 0.917 |
| Sowing rate : Mixed sowing¶ | –8.41 ± 17.22 | 20/16 | –0.49 | 0.632 |
| Sowing rate : With weed removal§ | –1.27 ± 13.23 | 20/17 | –0.10 | 0.925 |
| Mixed sowing¶ : With weed removal§ | 15.66 ± 27.74 | 20/17 | 0.56 | 0.580 |
| *Consolida regalis* |  |  |  |  |
| Intercept | –1.01 ± 8.88 | 19/16 | –0.11 | 0.911 |
| Sowing rate‡ | 14.24 ± 4.96 | 19/15 | 2.87 | 0.012 |
| Mixed sowing¶ | –1.37 ± 11.07 | 19/15 | –0.12 | 0.903 |
| With weed removal§ | 2.50 ± 10.77 | 19/16 | 0.23 | 0.820 |
| Sowing rate : Mixed sowing¶ | 3.29 ± 5.43 | 19/15 | 0.61 | 0.554 |
| Sowing rate : With weed removal§ | –0.89 ± 5.34 | 19/16 | –0.17 | 0.869 |
| Mixed sowing¶ : With weed removal§ | –0.85 ± 10.60 | 19/16 | –0.08 | 0.937 |
| *Lithospermum arvense* |  |  |  |  |
| Intercept | –10.73 ± 6.43 | 20/17 | –1.67 | 0.113 |
| Sowing rate‡ | 22.72 ± 3.46 | 20/16 | 6.57 | <0.001 |
| Mixed sowing¶ | –1.10 ± 8.80 | 20/16 | –0.12 | 0.902 |
| With weed removal§ | –0.13 ± 4.49 | 20/17 | –0.03 | 0.976 |
| Sowing rate : Mixed sowing¶ | 1.56 ± 4.65 | 20/16 | 0.34 | 0.741 |
| Sowing rate : With weed removal§ | 0.40 ± 2.13 | 20/17 | 0.19 | 0.851 |
| Mixed sowing¶ : With weed removal§ | 0.26 ± 4.45 | 20/17 | 0.06 | 0.954 |

*log10(x+1) transformed; †square root-transformed; ‡ log10 transformed; ¶ pure sowing as reference-level; § without wee removal as reference-level

**Figure S1.** Schematic illustration of the partial additive study design. Rye was sown in 40 plots with fixed sowing rate. Study species were added in ten sowing rates (compare Table 1) as single species (Leg = *Legousia speculum-veneris*, Con = *Consolida regalis*, Lit = *Lithospermum arvense*) or as a three-species mixture (mix).


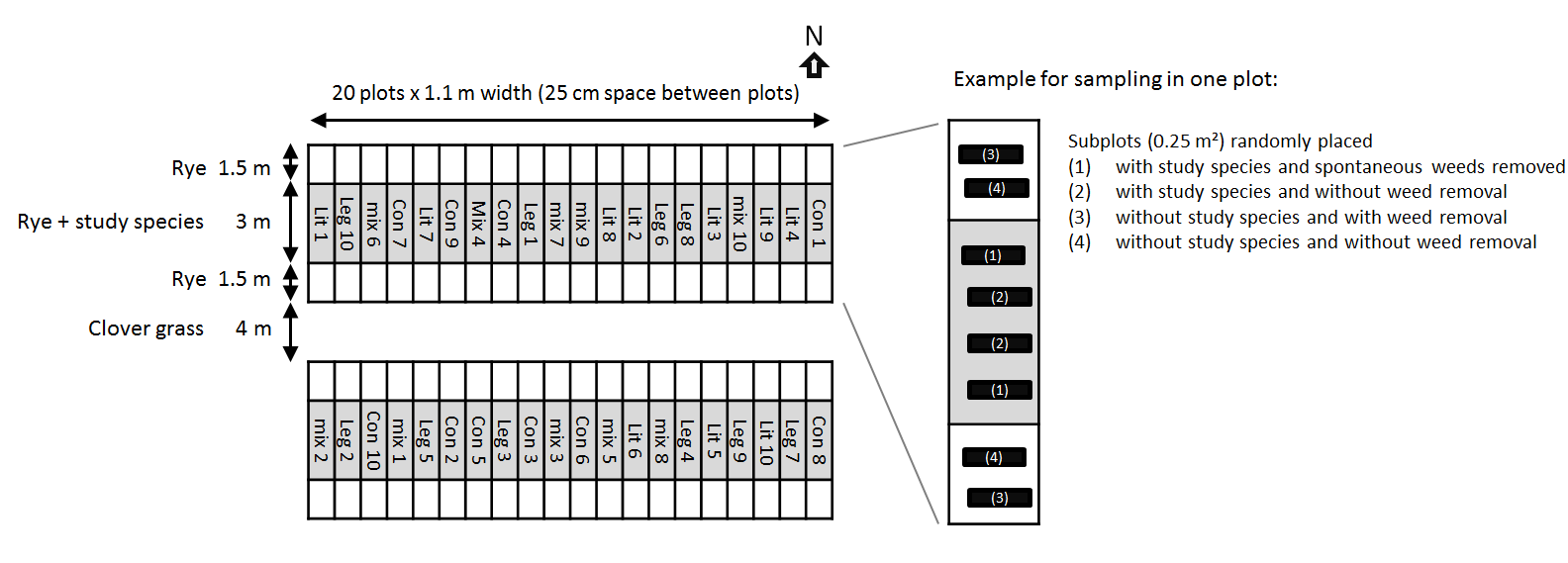

Supplement: Supplementary file 1 — Table S1. Establishment and reproduction of the study species Legousia speculum‐veneris (Leg), Consolida regalis (Con) and Lithospermum arvense (Lit) in pure and mixed sowing, with and without removal of spontaneous weeds. Table S2. Results of the full Linear Mixed‐Effects Models for establishment and seed production of the three re‐introduced arable plants at harvest time, with sowing rate, sowing type (pure and mixed sowing), weed removal (with and without removal of spontaneous weeds), and all two‐way interactions as explanatory variables. Figure S1. Schematic illustration of the partial additive study design. [file ECE3-6-5506-s001.docx]
